# Supplementary material for: PTEN and the PTEN-like phosphatase CnrN have both distinct and overlapping roles in a Dictyostelium chemorepulsion pathway
Source: J Cell Sci. 2024 Aug 1;137(15):jcs262054. doi: 10.1242/jcs.262054 (PMC11317092; doi:10.1242/jcs.262054)
Supplement: Supplementary information [file joces-137-262054-s1.pdf]

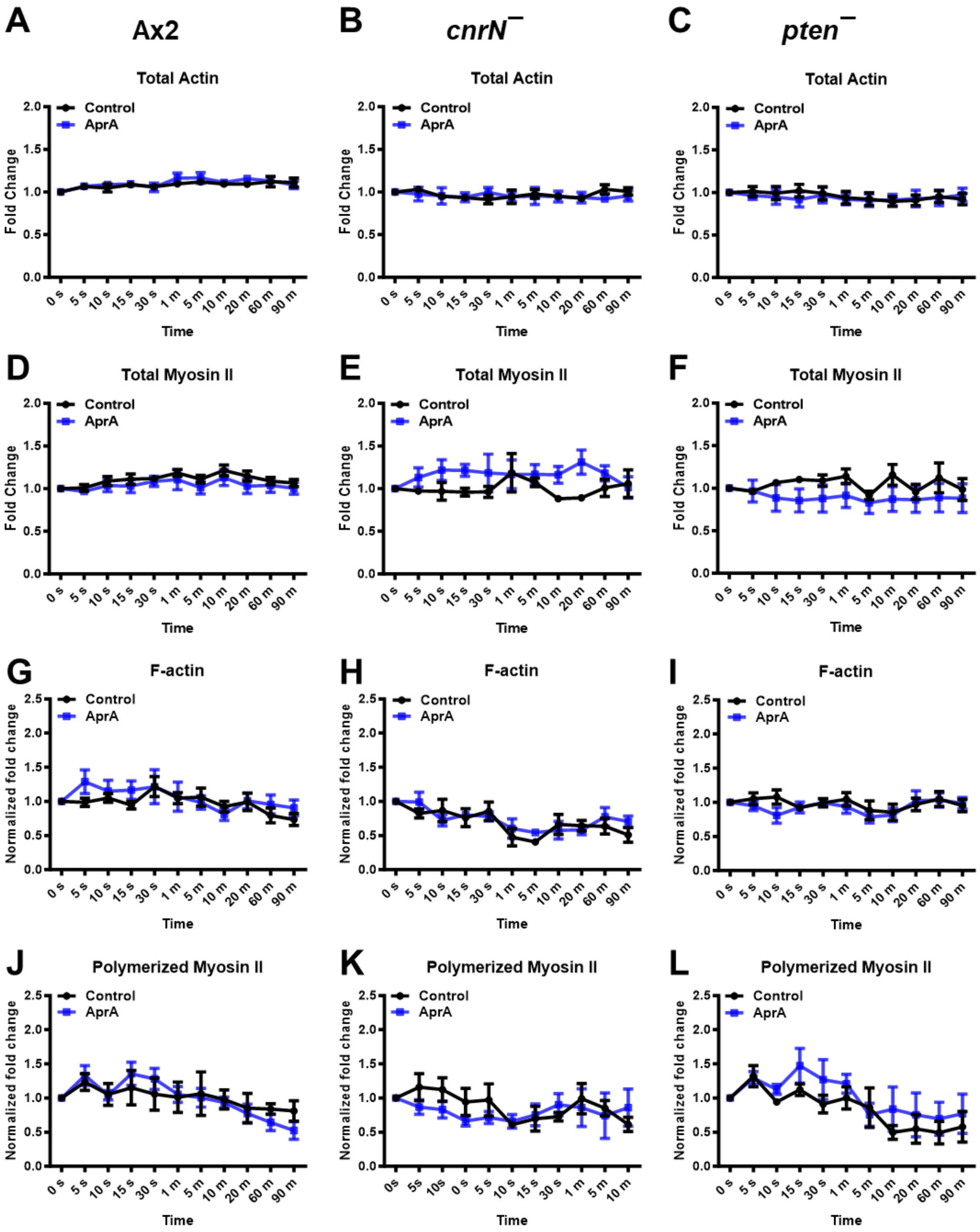
A
Ax2
B
cnrN⁻
C
pten⁻
Total Actin
Total Actin
Total Actin
D
Total Myosin II
E
Total Myosin II
F
Total Myosin II
G
F-actin
H
F-actin
I
F-actin
J
Polymerized Myosin II
K
Polymerized Myosin II
L
Polymerized Myosin II
Control
AprA
Fold Change
Normalized fold change
Time
0 s
5 s
10 s
15 s
30 s
1 m
5 m
10 m
20 m
60 m
90 m

Fig. S1. AprA does not alter total actin, total myosin II, F-actin, or polymerized myosin II in Ax2, *cnrN*¯, and *pten*¯ cells. (A – L) Total actin, total myosin II, F-actin, and polymerized myosin II levels in Ax2 (A, D, G, J), *cnrN*¯ (B, E, H, K), and *pten*¯ (C, F, I, L) cells incubated in growth medium (0 seconds) or in growth medium with 300 ng/ml AprA for the indicated times were estimated as described in Figure 2. Values are mean ± SEM for ≥ 3 independent experiments.

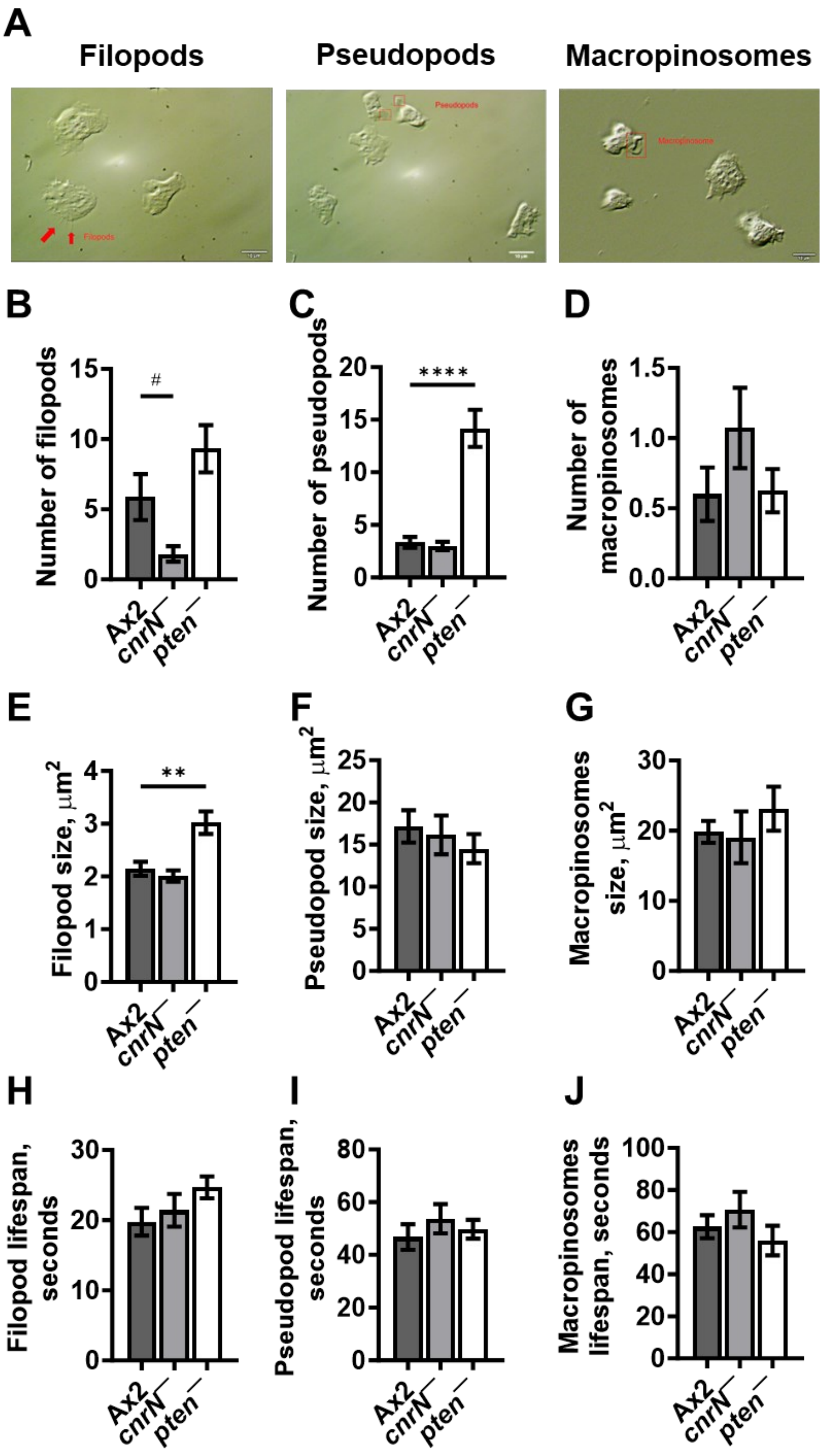

A
Filopods
Pseudopods
Macropinosomes
B
Number of filopods
#
C
Number of pseudopods
****
D
Number of macropinosomes
E
Filopod size, μm2
**
F
Pseudopod size, μm2
G
Macropinosomes size, μm2
H
Filopod lifespan, seconds
I
Pseudopod lifespan, seconds
J
Macropinosomes lifespan, seconds
Ax2
cnrN–
pten–

Fig. S2. *cnrN*$^{-}$ cells have inherently fewer filopods and *pten*$^{-}$ cells have inherently larger filopods and more pseudopods than Ax2 cells. Cells of the indicated strains in growth medium were allowed to adhere on a coverslip for 30 minutes. After adherence, images of cells were taken every 2 seconds for 5 minutes (A) and the number, sizes, and the apparent lifespan of filopods (A, E, H), pseudopods (C, F, I), and macropinosomes (D, G, J) were determined. Red arrows in A indicate filopods (left), and red boxes indicate pseudopods (middle) and macropinosomes (right). Values are mean ± SEM of > 18 cells from 3 independent experiments. $^{\#}$ $p < 0.01$ (Unpaired t test with Welch's correction) and * $p < 0.05$, ** $p < 0.01$, and **** $p < 0.0001$ (One-way ANOVA with Dunnett's multiple comparisons test).

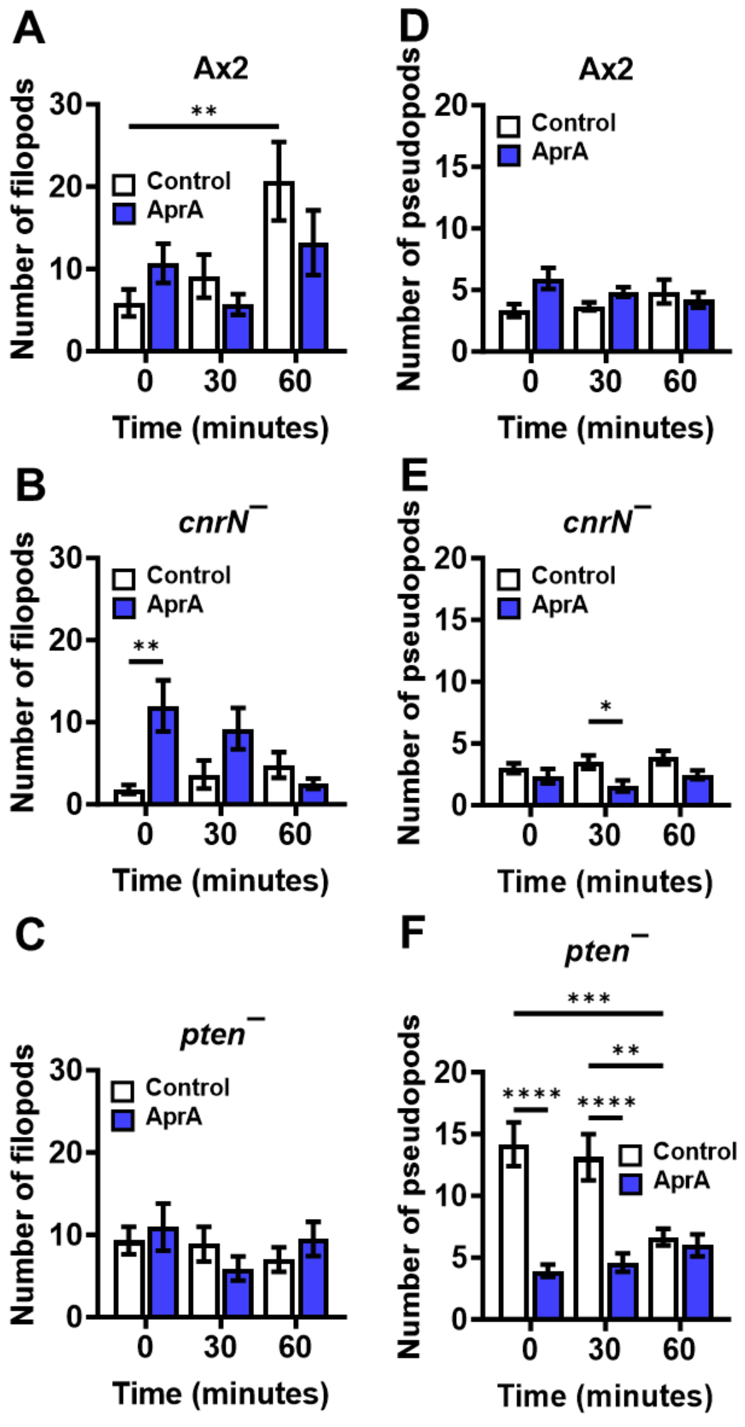

Journal of Cell Science • Supplementary information

Fig. S3. AprA effects on filopod, pseudopod, or macropinosome numbers in Ax2, *cnrN*$^{-}$, *pten*$^{-}$ cells. Cells of the indicated strains in growth medium were allowed to adhere on a petri dish for 30 minutes. After adherence, images of cells were taken at 0, 30 and 60 minutes, every 2 seconds for 5 minutes, to determine the number of filopods (A - C), pseudopods (D - F), and macropinosomes (G - I). Values are mean ± SEM of > 18 cells from 3 independent experiments. * $p < 0.05$, ** $p < 0.01$, *** $p < 0.001$, and **** $p < 0.0001$ (2way ANOVA with Tukey's multiple comparisons test).

Journal of Cell Science • Supplementary information

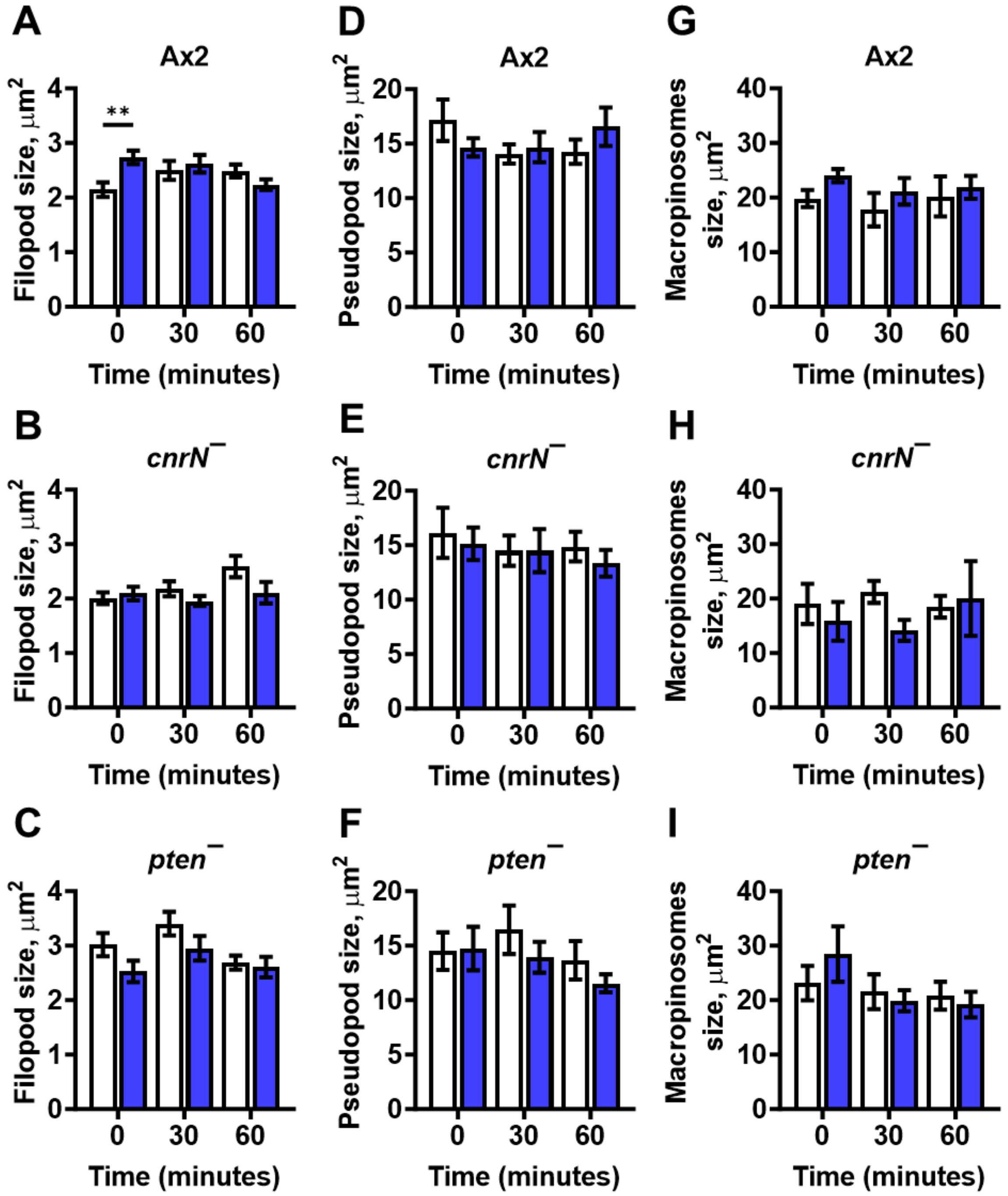

Fig. S4. AprA alters filopod sizes in Ax2, but not in *cnrN*⁻ and *pten*⁻ cells. Cells of the indicated strains in growth medium were allowed to adhere on a petri dish for 30 minutes. After adherence, images of cells were taken at 0, 30 and 60 minutes, every 2 seconds for 5 minutes, to determine the sizes of filopods (A - C), pseudopods (D - F), and macropinosomes (G - I). Values are mean ± SEM of > 18 cells from 3 independent experiments. ** $p < 0.01$ (2way ANOVA with Tukey's multiple comparisons test).

Journal of Cell Science • Supplementary information

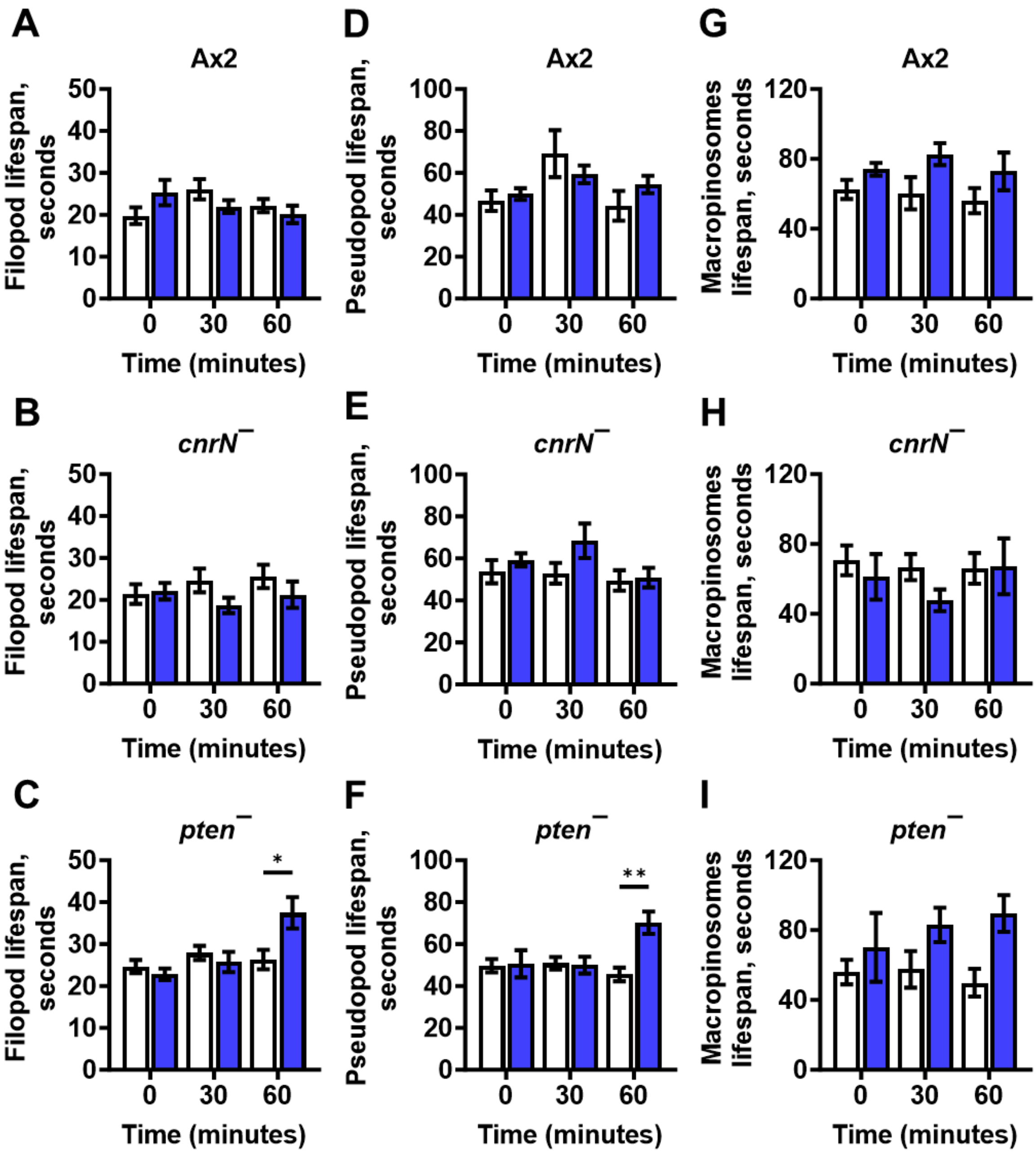

Fig. S5. AprA alters filopod and pseudopod lifespan in *pten*$^{-}$ cells, but not in Ax2 and *cnrN*$^{-}$ cells. Cells of the indicated strains in growth medium were allowed to adhere on a petri dish for 30 minutes. After adherence, images of cells were taken at 0, 30 and 60 minutes, every 2 seconds for 5 minutes, to determine the apparent lifespan of filopods (A - C), pseudopods (D - F), and macropinosomes (G - I). Values are mean ± SEM of > 18 cells from 3 independent experiments. * $p < 0.05$ (2way ANOVA with Tukey's multiple comparisons test).

Journal of Cell Science • Supplementary information
