# Supplementary material for: PTEN and the PTEN-like phosphatase CnrN have both distinct and overlapping roles in a Dictyostelium chemorepulsion pathway
Source: J Cell Sci. 2024 Aug 1;137(15):jcs262054. doi: 10.1242/jcs.262054 (PMC11317092; doi:10.1242/jcs.262054)
Supplement: Supplementary file 1 [file joces-137-262054_review_history.pdf]

PTEN and the PTEN-like phosphatase CnrN have both distinct and overlapping roles in a *Dictyostelium* chemorepulsion pathway

Kristen M. Consalvo, Ramesh Rijal, Steven Beruvides, Ryan Mitchell, Karissa Beauchemin, Danni Collins, Jack Scoggin, Jerome Scott and Richard H. Gomer
DOI: 10.1242/jcs.262054

Editor: Daniel Billadeau

Review timeline
Original submission: 23 February 2024
Editorial decision: 2 April 2024
First revision received: 15 May 2024
Accepted: 19 June 2024

Original submission

First decision letter

MS ID#: JOCES/2024/262054

MS TITLE: PTEN and the PTEN-like phosphatase CnrN have both distinct and overlapping roles in a Dictyostelium chemorepulsion pathway

AUTHORS: Kristen M. Consalvo, Ramesh Rijal, Steven Beruvides, Ryan Mitchell, Karissa Beauchemin, Danni Collins, Jack Scoggin, Jerome Scott, and Richard Gomer

We have now reached a decision on the above manuscript.

To see the reviewers' reports and a copy of this decision letter, please go to: https://submit-jcs.biologists.org and click on the 'Manuscripts with Decisions' queue in the Author Area. (Corresponding author only has access to reviews.)

As you will see, the reviewers were enthusiastic for this study, but raise several criticisms that prevent me from accepting the paper at this stage. In general, many of these are clarifications and edits in the document and Figure Legends. Reviewer 3 suggested that Supplemental Figure 1, become Figure 1 in the manuscript. I hope that you can make the requested changes and edits. I would be pleased to see a revised manuscript.

Please ensure that you clearly highlight all changes made in the revised manuscript. Please avoid using 'Tracked changes' in Word files as these are lost in PDF conversion.

I should be grateful if you would also provide a point-by-point response detailing how you have dealt with the points raised by the reviewers in the 'Response to Reviewers' box. Please attend to all of the reviewers' comments. If you do not agree with any of their criticisms or suggestions please explain clearly why this is so.

Reviewer 1

Advance summary and potential significance to field

© 2024. Published by The Company of Biologists under the terms of the Creative Commons Attribution License (https://creativecommons.org/licenses/by/4.0/).

The manuscript by Consalvo et al. describes the relationship between two phosphatidylinositol 3,4,5-trisphosphate (PIP3) phosphatases, PTEN and CnrN, and the AprA chemorepulsion pathway in Dictyostelium.
While many researchers in the field have focused their attention on positive chemotaxis (chemoattraction), Dr. Gomer and his group have introduced the notion of negative chemotaxis (chemorepulsion). In the past two decades, they have described numerous aspects of this interesting phenomenon and compared and contrasted it with chemoattraction. In this manuscript, they used the chemorepellent protein AprA as the central experimental modifier and tested the consequences of deletion of the PTEN and CnrN phosphatases on most of the known responses to AprA. They used methods in cell biology, microscopy and biochemistry to carefully document almost every aspect of the AprA pathway. The introduction section is clear and informative and provides all the necessary information for understanding the current work. The results section is a careful and methodical documentation of the data and the experimental methods and finding are state of the art. The discussion section is clear and well-written. It places the results in the broader context of the published literature and helps focus the interpretation of the data. There is an interesting theme of discussing the data from an evolutionary perspective, emphasizing the differences between the two enzymes and speculating on their function. Overall, this is a high-quality manuscript that is almost ready for publication. Below, I have listed a few items that would improve this manuscript even further.

Comments for the author

None of the issues listed below should prevent publication of the manuscript, but I hope the authors would consider them anyway.

Major items:

1. It is not clear from the abstract section what was known before this work was done, and which findings were discovered through the new experiments described here. A sentence should be added after the description of the previously known part, to explain what was the question that motivated this work followed by a phrase such as “Here, we show that ...”

2. Figure 1A shows that the levels of PI(4,5)P2 were higher in the pten- cells than in the Ax2 cells. This is unexpected based on the role of PTEN in the conversion of PI(3,4,5)P3 into PI(4,5)P2. Also in that figure expression of cnrN-gfp did not increase the basal levels of PI(4,5)P2, even though it should have. At the same time, the basal levels of PI(3,4,5)P3 behaved exactly as expected, suggesting that the enzymes were present and functional, and the detection methods were adequate. Could the authors please explain these unexpected results? A good place would be the paragraph that ends in line 140.

3. The summary of the experiments shown in Figure 1 is given in one sentence that spans lines 146-152.
This sentence is too long and convoluted. The authors are advised to break it up to shorter and clearer statements. Most importantly, this intermediate summary should state that the effect of AprA on the levels of PI(4,5)P2 depend on CnrN and not on PTEN, whereas the effect of AprA on the levels of and PI(3,4,5)P3 depend on both CnrN and PTEN.

4. The effect of AprA on cell proliferation, shown in Supplemental Figure 2F, is not very convincing. The effect of AprA on the wild-type proliferation is rather small, albeit statistically significant. The effect of AprA on the two mutants (cnrN- and pten-) is about the same as it is on the wild type, but it is not statistically significant. The problem with this finding is that both mutant strains exhibit a twofold reduction in proliferation rates compared to the wild type. Isn't it possible that this reduction obscures the effect of AprA on proliferation? It would be pertinent to mention this possibility in the text.

5. The authors say in the figure legend to Supplemental Figure 2F that values are mean and SEM form more than 10 cells per strain. Presumably, the term 'cells' here means plates or chambers of a multititer plate, whereas it means live Dictyostelium cells everywhere else in the manuscript. Searching the methods section did not help clarify this issue because there is no description there (or in the reference to Choe et al. 2009) of how the cells were incubated.

© 2024. Published by The Company of Biologists under the terms of the Creative Commons Attribution License (https://creativecommons.org/licenses/by/4.0/).

6. The last results section, starting in line 216, is rather unusual. First, the section relies on four supplemental figures - equal to the total number of main figures and more than all the other supplemental figures combined. Second, it provides a small number of experiments that indicate relevant differences embedded in a long list of experiments that mostly show no difference. While the comprehensive nature of the experiments and the internal consistency of the data are compelling, it is hard to see the forest for the trees here. It might be good if the authors could summarize the most relevant differences in a table or some illustration in the main text, while leaving the supplemental figures as they are, for completeness.

7. The summary of the last set of experiments is given in one sentence that spans lines 259-265. This sentence is too long and convoluted. The authors are advised to break it up to shorter and clearer statements.

Minor items:

1. Line 77 – delete the word 'at'

2. Line 377 – should 'immunofluorescence' be replaced with 'fluorescence'?

3. Line 605 – the term 'equivalent volume of buffer' is unclear. Presumably, this treatment defines the basal levels. The authors should state that in the figure legend and/or in the text (line 131), to avoid ambiguity.

4. In the PDF version I received, many of the 'greater than', 'smaller than' or 'equals' symbols have been presumably replaced with empty squares (e.g. p values in lines 609 and 623.

<u>Reviewer 2</u>

Advance summary and potential significance to field

The authors report observations suggesting that the Dictyostelium PTEN-like phosphatase CnrN has some roles in regulating chemorepulsion in this organism that are distinct from those of PTEN while other roles are redundant. This is interesting from an evolutionary perspective because this CnrN phosphatase is lost in higher eukaryotes, and also because it highlights the usefulness of Dictyostelium to decipher the different roles of PTEN and PIP3 metabolism in biology, roles that have merged in higher eukaryotes and make it more difficult to study process-specific mechanisms.

Comments for the author

The study conclusions are well supported by the presented data. However, I would encourage some revisions that I believe would improve the manuscript:

- Sometimes the data show differences but that weren't found to be statistically significant with the tests used. The authors should then specify in their results descriptions when that is the case and that they conclude that there is no (or there is) significant difference. For example, AprA seems to have reproducible lowering effects on PIP3 levels in PTEN null cells (Fig. 1I). The fact that it wasn't found to be statistically significant doesn't necessarily mean the effects are not real and should perhaps be considered and discussed. Same consideration should be made about the active Ras data in Fig. 3E.
- Fig. 1A shows higher PIP2 levels in both PTEN null and PTEN null expressing PTEN-GFP, which should theoretically rescue the PTEN null phenotype. The authors should provide an explanation for these unintuitive observations, in the result section, and refer to further discussions in the discussion section if needed.
- Fig. 2A and 2B, the authors need to explain or provide a control for how they can be certain that the bands they are quantifying from whole cell lysates Coomassie staining are actin and myosin. This method of quantifying these cytoskeletal proteins is not ideal and the authors should do western blotting instead, because in a total cell lysate many proteins migrate with the same apparent molecular weight.

© 2024. Published by The Company of Biologists under the terms of the Creative Commons Attribution License (https://creativecommons.org/licenses/by/4.0/).

- Fig. 3, the authors need to explain how they normalize the total Ras protein to total protein content.
- The authors should show all data points in all graphs to clearly show the spread in the data.
- Many of the data currently presented in supplementary figures should be included in main figures as they are key to the conclusions of the study, including S2, S4, S5, and S7.
- It would help the reader if the authors added clear conclusions about the reported observations at the end of each section instead of just reiterating the results in (very) long sentences that are hard to read.

Reviewer 3

Advance summary and potential significance to field

The manuscript by Consalvo et al entitled PTEN and the PTEN-like phosphatase CnrN have both distinct and overlapping roles in a Dictyostelium chemorepulsion pathway sets out to examine how these two phosphatases regulate responses to the chemorepellent AprA. The authors attempt to determine how these two lipid phosphatases work when cells are put under the control of a chemorepellent. Understanding the mechanism regulating this process would be of interest to many in the directed cell migration field.

Comments for the author

By examining a number of different cellular responses, the authors conclude that the two phosphatases work on different membrane domains. A considerable amount of work went into these studies, but the story is somewhat lost after figure 3 and the reviewer suggests the work be revised.

Major Comments:
The results section starts by going right to the supplemental figures, and these results are critical for a major finding of the paper. This data should really be in Figure 1. The authors should clearly demonstrate that PTEN localizes towards the AprA gradient. Furthermore, it has been well established that PTEN and Ras/PI3K are reciprocally regulated, so showing Ras and PI3K activity on the low side of the chemorepellent gradient would also be warranted. Better images and or including videos would strengthen these results. However, it is important to point out in the writing that you are imaging vegetative cells, which are unpolarized, and undergoing macropinocytosis, so they will not form a distinct front and rear as starving cells do. That means that the localization of PTEN or PLCD to the high side of the chemorepellent will not be as localized as has been published for highly motile, polarized cells (which also lack macropinosomes). But that is OK and would be expected.

Figure 1A : the authors show that there is ~10 times increase in basal PI(4,5)P2 level in pten null cells compared to Ax2 cells and 20X in pten rescued cells. It is surprising that the difference is so large. It would also be expected that there would be slightly less PI(4,5)P2 since you no longer are able to convert PIP3 to PI(4,5)P2, which is the opposite of what the authors show. If this is indeed the case, please provide some rationale for why the PI(4,5)P2 goes up. Another issue is that one would not expect the inability to regulate PI(3,4,5)P3 to have much of an effect on PI(4,5)P2 levels because it only makes up a small fraction of total PIP levels and does not have much effect on the bulk levels of PI(4,5)P2, since they are so much larger. The authors do show, that overall, PI(3,4,5)P3 levels are lower, which would be expected. Could the authors offer a positive control for their ELISA assay? The authors could stimulate developed cells with cAMP and perform the ELISA, but I would check the literature first to see if that even leads to much of a change in overall PI(4,5)P2 levels (see Clark et al EMBO 2014 for some discussion. PMCID: PMC4282506). However, PI(3,4,5)P3 levels should change. The authors could use a PI(4,5)P2-specific biosensor (such as PLCdelta) or PHAKT (for PIP3) assay in their cells to validate this, or at least offer a sound explanation.

Figure 1E,G,J,L : It is not clear why the other timepoints are not shown for those conditions? Please comment.

© 2024. Published by The Company of Biologists under the terms of the Creative Commons Attribution License (https://creativecommons.org/licenses/by/4.0/).

Figure 2B: The Y-axis is not labelled.

Figure 2A-E. Maybe I missed it, but are the lanes in the Coomassie and Western blots from the same or independent experiments? Please comment.

Line 179-181. It is stated that cnrR is needed for Myosin dephosphorylation after AprA treatment because there is no reduction in phosphorylation vs baseline in cnrR- cells (Fig 2G). However, cnrR- cells already have greatly reduced Myosin phosphorylation (Fig. 2E). How do we know that cells are capable of further reducing phosphorylation levels at all from this reduced baseline?

Figure 3: All bar graphs could use individual data points to assess the distribution of the data, (like in figure 2) especially in Fig 3E where it really looks like there might be a reduction in active Ras levels.

Figure 3: Why are there not error bars on any of the initial conditions (eg Ax2 in 3B). I understand that the other values are normalized to AX2, however if there are 3 independent experiments, each AX2 individual experiment would likely differ from that average.

Supplementary Figure 1: the DNA ladders should be marked properly

Also, for Figures S4, S5, etc., the authors would do well to include some imaging data to supplement these graphs. Additionally, while there is some description of how filopodia, macropinosomes, and pseudopods are quantified, it isn't entirely clear how one distinguishes a macropinosome from a pseudopod. It wasn't clear why there would be such a large change in the size of your pseudopods here compared to what you previously reported (10X larger!, line 233). Without 3D imaging, it is difficult to makes these comparisons. If you are going to include this data, Please elaborate in the methods section how these parameters were scored and measured.

Minor Comments and Typos:
There are many run-on sentences in the manuscript, I mentioned several of them below.
Line 52: 'phosphatidylinositol3OH -kinases' is not written correctly Line 77: Delete the extra 'At'
Line 146-152 is a run-on sentence. Please break those conclusions up.
Line 222 cells have "a" slow Line 224 To determine (sentence is a run-on and some of that should be in methods section).
Line 306: This increase…(run-on sentence).
Line 353: In support of….(run-on sentence).
Fix Veltman 2015 Reference. Biochem Soc Trans (2015) 43 (1): 129–132. PMID: 25619258. It is important to understand the conclusions about macropinocytosis and PIP3 in this paper as well and their primary research papers. They were comparing NC4 cell lines (which grow on bacteria) with the AX2 and 3 lines, which have a mutation that upregulates macropinocytosis and PIP3, thus allowing the cells to grow axenically.

First revision

Author response to reviewers' comments

Reviewer 1 Advance Summary and Potential Significance to Field...
The manuscript by Consalvo et al. describes the relationship between two phosphatidylinositol 3,4,5-trisphosphate (PIP3) phosphatases, PTEN and CnrN, and the AprA chemorepulsion pathway in Dictyostelium. While many researchers in the field have focused their attention on positive chemotaxis (chemoattraction), Dr. Gomer and his group have introduced the notion of negative chemotaxis (chemorepulsion). In the past two decades, they have described numerous aspects of this interesting phenomenon and compared and contrasted it with chemoattraction. In this manuscript, they used the chemorepellent protein AprA as the central experimental modifier and tested the consequences of deletion of the PTEN and CnrN phosphatases on most of the known responses to AprA. They used methods in cell biology, microscopy and biochemistry to carefully

© 2024. Published by The Company of Biologists under the terms of the Creative Commons Attribution License (https://creativecommons.org/licenses/by/4.0/).

document almost every aspect of the AprA pathway. The introduction section is clear and informative and provides all the necessary information for understanding the current work. The results section is a careful and methodical documentation of the data and the experimental methods and finding are state of the art. The discussion section is clear and well-written. It places the results in the broader context of the published literature and helps focus the interpretation of the data. There is an interesting theme of discussing the data from an evolutionary perspective, emphasizing the differences between the two enzymes and speculating on their function. Overall, this is a high-quality manuscript that is almost ready for publication. Below, I have listed a few items that would improve this manuscript even further.

Reviewer 1 Comments for the Author...
None of the issues listed below should prevent the publication of the manuscript, but I hope the authors would consider them anyway.

Major items:
1. It is not clear from the abstract section what was known before this work was done, and which findings were discovered through the new experiments described here. A sentence should be added after the description of the previously known part, to explain what was the question that motivated this work, followed by a phrase such as "Here, we show that ..."
We have now shortened the abstract following the JCS guidelines and added a sentence in the abstract stating the question that motivated this work as: "Little is known about eukaryotic chemorepulsion. The enzymes Phosphatase and tensin homolog (PTEN) and CnrN dephosphorylate phosphatidylinositol 3,4,5-trisphosphate [PI(3,4,5)P3] to phosphatidylinositol 4,5-bisphosphate [PI(4,5)P2]. Dictyostelium discoideum cells require both PTEN and CnrN to induce chemorepulsion of cells away from the secreted chemorepellent protein AprA. How D. discoideum cells utilize two proteins with redundant phosphatase activities in response to AprA is unclear. Here, we show that D. discoideum cells require both PTEN and CnrN to locally inhibit Ras activation, decrease basal levels of PI(3,4,5)P3, and increase basal numbers of macropinosomes, and AprA prevents this increase. AprA requires both PTEN and CnrN to increase PI(4,5)P2 levels, decrease PI(3,4,5)P3 levels, inhibit proliferation, decrease myosin II phosphorylation, and increase filopod sizes. PTEN, but not CnrN, decreases basal levels of PI(4,5)P2, and AprA requires PTEN, but not CnrN, to induce cell roundness. Together, our results suggest that CnrN and PTEN play unique roles in AprA-induced chemorepulsion."

2. Figure 1A shows that the levels of PI(4,5)P2 were higher in the pten- cells than in the Ax2 cells. This is unexpected based on the role of PTEN in the conversion of PI(3,4,5)P3 into PI(4,5)P2. Also in that figure, expression of cnrN-gfp did not increase the basal levels of PI(4,5)P2, even though it should have. At the same time, the basal levels of PI(3,4,5)P3 behaved exactly as expected, suggesting that the enzymes were present and functional, and the detection methods were adequate. Could the authors please explain these unexpected results? A good place would be the paragraph that ends in line 140.

Thank you for the suggestion, but we have already discussed why levels of PI(4,5)P2 were higher in the pten- cells than in the Ax2 cells in the discussion section. We have now included why the expression of CnrN-GFP did not alter the levels of PI(4,5)P2 in line 292 - 296 as: "Loss or overexpression of PTEN but not CnrN increased basal PI(4,5)P2 levels, possibly due to dysregulation of CnrN activity as a result of either loss of PTEN or overexpression of PTEN in cells lacking PTEN and optimum PTEN levels appears to be necessary to maintain basal PI(4,5)P2 levels. Loss of either PTEN or CnrN increased basal PI(3,4,5)P3 levels, indicating that both PTEN and CnrN are required to maintain the basal PI(3,4,5)P3 levels."

3. The summary of the experiments shown in Figure 1 is given in one sentence that spans lines 146-152. This sentence is too long and convoluted. The authors are advised to break it up to shorter and clearer statements. Most importantly, this intermediate summary should state that the effect of AprA on the levels of PI(4,5)P2 depend on CnrN and not on PTEN, whereas the effect of AprA on the levels of and PI(3,4,5)P3 depend on both CnrN and PTEN.

We have now carefully made clearer statements of the summary of the experiments as suggested in lines 158 - 164 as "Together, these data indicate that PTEN but not CnrN decreases basal levels of PI(4,5)P2. Assuming that the PTEN levels in pten¯/pten-gfp are not exactly the same as in Ax2 cells, the proper levels of PTEN are needed to maintain basal levels of PI(4,5)P2. Both PTEN and CnrN decrease basal levels of PI(3,4,5)P3. The effect of AprA on the levels of PI(4,5)P2

© 2024. Published by The Company of Biologists under the terms of the Creative Commons Attribution License (https://creativecommons.org/licenses/by/4.0/).

depends on CnrN and not on PTEN. In the absence of PTEN but not CnrN, AprA causes an unknown enzyme to decrease levels of PI(4,5)P2. The effect of AprA on the levels of PI(3,4,5)P3 depend on both CnrN and PTEN."

4. The effect of AprA on cell proliferation, shown in Supplemental Figure 2F, is not very convincing. The effect of AprA on the wild-type proliferation is rather small, albeit statistically significant. The effect of AprA on the two mutants (cnrN- and pten-) is about the same as it is on the wild type, but it is not statistically significant. The problem with this finding is that both mutant strains exhibit a twofold reduction in proliferation rates compared to the wild type. Isn't it possible that this reduction obscures the effect of AprA on proliferation? It would be pertinent to mention this possibility in the text.

We have now mentioned in lines 122 – 125 as: "It is possible that the effect of AprA on the proliferation of cnrN¯ or pten¯ cells is obscured due to the already ~ two-fold reduced rate of proliferation of cnrN¯ or pten¯ cells compared to Ax2. Together, these data suggest that AprA may require both CnrN and PTEN to inhibit proliferation."

5. The authors say in the figure legend to Supplemental Figure 2F that values are mean and SEM form more than 10 cells per strain. Presumably, the term 'cells' here means plates or chambers of a multititer plate, whereas it means live Dictyostelium cells everywhere else in the manuscript. Searching the methods section did not help clarify this issue because there is no description there (or in the reference to Choe et al. 2009) of how the cells were incubated.

Thanks for catching this. The proliferation of cells was monitored by measuring the cell density using a hemacytometer at the indicated times, and the percentage proliferation was calculated by dividing the cell density on day 1 by the cell density on day 0. We have now clarified the text in the figure legend and carefully explained it in the method section.

6. The last results section, starting in line 216, is rather unusual. First, the section relies on four supplemental figures - equal to the total number of main figures and more than all the other supplemental figures combined. Second, it provides a small number of experiments that indicate relevant differences, embedded in a long list of experiments that mostly show no difference. While the comprehensive nature of the experiments and the internal consistency of the data are compelling, it is hard to see the forest for the trees here. It might be good if the authors could summarize the most relevant differences in a table or some illustration in the main text, while leaving the supplemental figures as they are, for completeness.

Thank you for the suggestions. Due to the exploratory nature of the experiments, we have opted to include all results, even if only a small number of experiments indicate significant differences. Following advice from Reviewer 2, we have now diligently incorporated clear conclusions regarding the most relevant differences at the conclusion of each section.

7. The summary of the last set of experiments is given in one sentence that spans lines 259-265. This sentence is too long and convoluted. The authors are advised to break it up to shorter and clearer statements.

We have now carefully made clearer statements of the summary of the experiments for each section of the results as suggested.

Minor items:

8. Line 77 – delete the word 'at'

Done

9. Line 377 – should 'immunofluorescence' be replaced with 'fluorescence'?

In line 377 (now 378), immunofluorescence is now replaced with fluorescence.

10. Line 605 – the term 'equivalent volume of buffer' is unclear. Presumably, this treatment defines the basal levels. The authors should state that in the figure legend and/or in the text (line 131), to avoid ambiguity.

Thanks for catching this. We have now carefully stated in line 646 that "Cells were incubated in HL5 with 300 ng/ml AprA for the indicated amounts of time or an equivalent volume of 20 mM NaPO4, pH 6.3 buffer for 0 s (control)" in the figure legend and in line 142 "…were exposed to a uniform concentration of AprA for the indicated amounts of time or an equivalent volume of 20 mM NaPO4, pH 6.3 buffer for 0 s (control)."

© 2024. Published by The Company of Biologists under the terms of the Creative Commons Attribution License (https://creativecommons.org/licenses/by/4.0/).

11. In the PDF version I received, many of the ‘greater than’, ‘smaller than’ or ‘equals’ symbols have been presumably replaced with empty squares (e.g. p values in lines 609 and 623.

The PDF conversion of the Word file has altered the symbols. We have now carefully checked that the symbols are correct in the text.

Reviewer 2 Advance Summary and Potential Significance to Field...
The authors report observations suggesting that the Dictyostelium PTEN-like phosphatase CnrN has some roles in regulating chemorepulsion in this organism that are distinct from those of PTEN while other roles are redundant. This is interesting from an evolutionary perspective because this CnrN phosphatase is lost in higher eukaryotes, and also because it highlights the usefulness of Dictyostelium to decipher the different roles of PTEN and PIP3 metabolism in biology, roles that have merged in higher eukaryotes and make it more difficult to study process-specific mechanisms.

Reviewer 2 Comments for the Author...
The study conclusions are well supported by the presented data. However, I would encourage some revisions that I believe would improve the manuscript:

1. Sometimes the data show differences but that weren’t found to be statistically significant with the tests used. The authors should then specify in their results descriptions when that is the case and that they conclude that there is no (or there is) significant difference. For example, AprA seems to have reproducible lowering effects on PIP3 levels in PTEN null cells (Fig. 1I). The fact that it wasn’t found to be statistically significant doesn’t necessarily mean the effects are not real and should perhaps be considered and discussed. Same consideration should be made about the active Ras data in Fig. 3E.

We appreciate your suggestions. Although AprA may have subtle effects on PIP3 levels in PTEN null cells and active Ras, compared to the control, the effects of AprA on PIP3 and active-Ras levels were consistent across all biological replicates. We have consistently considered biological effects relevant when the p-value is < 0.05, following our standard procedure for many years.

2. Fig. 1A shows higher PIP2 levels in both PTEN null and PTEN null expressing PTEN-GFP, which should theoretically rescue the PTEN null phenotype. The authors should provide an explanation for these unintuitive observations, in the result section, and refer to further discussions in the discussion section if needed.

We have now clarified carefully why PIP2 levels are elevated in PTEN-null and PTEN null expressing PTEN-GFP cells in the discussion section from lines 292 – 296 as “Loss or overexpression of PTEN but not CnrN increased basal PI(4,5)P2 levels, possibly due to dysregulation of CnrN activity as a result of either loss of PTEN or overexpression of PTEN in cells lacking PTEN and optimum PTEN levels appears to be necessary to maintain basal PI(4,5)P2 levels. Loss of either PTEN or CnrN increased basal PI(3,4,5)P3 levels, indicating that both PTEN and CnrN are required to maintain the basal PI(3,4,5)P3 levels.”

3. Fig. 2A and 2B, the authors need to explain or provide a control for how they can be certain that the bands they are quantifying from whole cell lysates Coomassie staining are actin and myosin. This method of quantifying these cytoskeletal proteins is not ideal and the authors should do western blotting instead, because in a total cell lysate many proteins migrate with the same apparent molecular weight.

We have now repeated actin and myosin quantification by western blotting using anti-actin and anti-myosin antibodies. Compared to the quantification of actin and myosin from the whole cell lysates by Coomassie staining, the levels of total actin and myosin in cells lacking CnrN or PTEN was not significant compared to Ax2 when measured by densitometric analysis of the western blots. We apologize for these inconsistencies. Figures 2A and B (now Figures 4A and B) are now replaced with new figures, figure legends are updated, and the results are corrected in line 174 as “Both cnrN¯ and pten¯ cells had normal basal total actin and myosin II levels (Figures 4A and B).”

4. Fig. 3, the authors need to explain how they normalize the total Ras protein to total protein content.

We have carefully explained how the total Ras protein was normalized to total protein content in the method section from lines 462 - 469 as: “Total cell lysates or Raf-RBD affinity bead

© 2024. Published by The Company of Biologists under the terms of the Creative Commons Attribution License (https://creativecommons.org/licenses/by/4.0/).

pull-down samples were run on SDS-polyacrylamide gels, western blots of the gels were stained with anti-Pan Ras antibodies, and densitometry was used to estimate levels of Ras in the Raf-RBD affinity bead pull-down assays. The protein content in the total cell lysates was estimated using a BCA protein assay kit (Cat#23227, Thermo Scientific, Rockford, IL) following the manufacturer's instructions. For an accurate comparison of Ras levels in all samples, Ras levels from densitometric analysis of western blots were normalized by dividing Ras levels by the protein content in the total cell lysates."

5. The authors should show all data points in all graphs to clearly show the spread in the data.

We have now shown all the data points to clearly show the spread in the data.

6. Many of the data currently presented in supplementary figures should be included in the main figures as they are key to the conclusions of the study, including S2, S4, S5, and S7.

Thank you for the suggestion. We have now included Figure S2 as Figure 2. However, we agree with Reviewer 1's recommendation to maintain the supplementary figures S4 – S7 (now S3 - S6) as they are.

7. It would help the reader if the authors added clear conclusions about the reported observations at the end of each section instead of just reiterating the results in (very) long sentences that are hard to read.

We have now carefully added clear conclusions at the end of each section.

Reviewer 3 Advance Summary and Potential Significance to Field...
The manuscript by Consalvo et al entitled PTEN and the PTEN-like phosphatase CnrN have both distinct and overlapping roles in a Dictyostelium chemorepulsion pathway sets out to examine how these two phosphatases regulate responses to the chemorepellent AprA. The authors attempt to determine how these two lipid phosphatases work when cells are put under the control of a chemorepellent. Understanding the mechanism regulating this process would be of interest to many in the directed cell migration field.

Reviewer 3 Comments for the Author...
By examining a number of different cellular responses, the authors conclude that the two phosphatases work on different membrane domains. A considerable amount of work went into these studies, but the story is somewhat lost after figure 3 and the reviewer suggests the work be revised.

Major Comments:
1. The results section starts by going right to the supplemental figures, and these results are critical for a major finding of the paper. This data should really be in Figure 1.

We have now made Supplementary Figures 1 and 2 as Figure 1 and Figure 2, respectively, and adjusted the remaining figures, figure legends, and citations in the text accordingly.

2. The authors should clearly demonstrate that PTEN localizes towards the AprA gradient. Furthermore, it has been well established that PTEN and Ras/PI3K are reciprocally regulated, so showing Ras and PI3K activity on the low side of the chemorepellent gradient would also be warranted.

We do appreciate your suggestions for testing Ras and PI3K activity. Demonstrating distinct PTEN and Ras/PI3K localizations on opposite sides of the cell within the AprA gradient may not be necessary, given the comprehensive evidence from our previous publications. We have now added a paragraph in the discussion section as "Our previous work has established that PI3K is not needed during AprA mediated chemorepulsion (Phillips and Gomer, 2012), and it does not affect total F-actin levels during chemorepulsion. Instead, it induces F-actin and active Ras binding RBD-Raf1-GFP localization towards the side of the cell opposite to the AprA source (Kirolos and Gomer, 2022; Rijal et al., 2019). Building upon those previous findings, PTEN-GFP localization at the side of cells facing in an AprA gradient suggests a reciprocal regulation mechanism between PTEN and Ras.

3. Better images and or including videos would strengthen these results. However, it is important to point out in the writing that you are imaging vegetative cells, which are unpolarized, and undergoing macropinocytosis, so they will not form a distinct front and rear as starving cells

© 2024. Published by The Company of Biologists under the terms of the Creative Commons Attribution License (https://creativecommons.org/licenses/by/4.0/).

do. That means that the localization of PTEN or PLCD to the high side of the chemorepellent will not be as localized as has been published for highly motile, polarized cells (which also lack macropinosomes). But that is OK and would be expected.

Videos could really help show our findings better. However, due to background fluorescent signals originating from the HL5 media, we did not examine PTEN-GFP localization in live cells in the AprA gradient. To reduce the issue of background fluorescence, we fixed the cells after exposing cells to the AprA gradient and then analyzed PTEN-GFP localization, as we explained in the method.

We agree that the localization of PTEN in cells in an AprA gradient may not be as localized as has been published for highly motile, polarized cells.

4. Figure 1A : the authors show that there is ~10 times increase in basal PI(4,5)P2 level in pten null cells compared to Ax2 cells and 20X in pten rescued cells. It is surprising that the difference is so large. It would also be expected that there would be slightly less PI(4,5)P2 since you no longer are able to convert PIP3 to PI(4,5)P2, which is the opposite of what the authors show. If this is indeed the case, please provide some rationale for why the PI(4,5)P2 goes up. Another issue is that one would not expect the inability to regulate PI(3,4,5)P3 to have much of an effect on PI(4,5)P2 levels because it only makes up a small fraction of total PIP levels and does not have much effect on the bulk levels of PI(4,5)P2, since they are so much larger. The authors do show, that overall, PI(3,4,5)P3 levels are lower, which would be expected. Could the authors offer a positive control for their ELISA assay? The authors could stimulate developed cells with cAMP and perform the ELISA, but I would check the literature first to see if that even leads to much of a change in overall PI(4,5)P2 levels (see Clark et al EMBO 2014 for some discussion. PMCID: PMC4282506). However, PI(3,4,5)P3 levels should change. The authors could use a PI(4,5)P2-specific biosensor (such as PLCdelta) or PHAKT (for PIP3) assay in their cells to validate this, or at least offer a sound explanation.

We have carefully discussed why altering PTEN, but not CnrN, leads to an increase in basal PI(4,5)P2 levels, as detailed in the discussion section from lines 292 to 296. Indeed, we were surprised to observe a significant impact on PI(4,5)P2 levels when PI(3,4,5)P3 levels were increased, particularly in cells with altered PTEN expression.

The PI(4,5)P2 and PI(3,4,5)P3 of known concentrations provided in the kit were used as positive controls. We have now explained in the method section in line 426.

We appreciate your suggestions. Cells stimulated with cAMP would be a negative control for PIP3. Clark et al EMBO 2014 showed that cAMP increases levels of PIP3 from 5 seconds to more than 10 minutes and drops the PIP3 levels to normal after 20 minutes, without significantly altering the levels of PIP2. We have now elaborated on this in lines 151 – 153 as “cAMP stimulation of Dictyostelium cells causes an increase in the levels of PIP3 (as indicated in the paper) as early as 5 seconds, and that level of PIP3 dropped to normal after 20 minutes, but does not alter the levels of PIP2 (Clark et al., 2014). In contrast, AprA caused a decrease in PI(3,4,5)P3 levels at 10 and 20 minutes in Ax2 cells (Figure 3H).”

5. Figure 1E,G,J,L : It is not clear why the other time points are not shown for those conditions. Please comment.

We have now clarified in the results section why we omitted other time points in Figures 1E, G, J, and L (now updated to Figures 3E, G, J, and L), as described from lines 142 to 145 as “AprA increased PI(4,5)P2 levels at 5 and 10 minutes in Ax2 cells (Figure 3C), and that level was maximum at 10 minutes. Therefore, we opted to expose pten¯ or cnrN¯ cells overexpressing PTEN-GFP or CnrN-GFP to AprA for 10 minutes.”

6. Figure 2B: The Y-axis is not labelled.

Thanks for catching this. The Y-axis is labeled now (now Figure 4B).

7. Figure 2A-E. Maybe I missed it, but are the lanes in the Coomassie and Western blots from the same or independent experiments? Please comment.

Yes, the lanes in both the Coomassie and Western blots are from the same experiments. We have now thoroughly revised the legend for Figure 2 (now Figure 4) to provide clear clarification.

© 2024. Published by The Company of Biologists under the terms of the Creative Commons Attribution License (https://creativecommons.org/licenses/by/4.0/).

8. Line 179-181. It is stated that cnrR is needed for Myosin dephosphorylation after AprA treatment because there is no reduction in phosphorylation vs baseline in cnrR- cells (Fig 2G). However, cnrR- cells already have greatly reduced Myosin phosphorylation (Fig. 2E). How do we know that cells are capable of further reducing phosphorylation levels at all from this reduced baseline?

It is possible that the observed baseline level does not represent the lowest achievable level of dephosphorylation. However, we agree with your point that we cannot definitively conclude whether cells are capable of further reducing phosphorylation levels from the already reduced baseline observed in cnrN- cells.

9. Figure 3: All bar graphs could use individual data points to assess the distribution of the data, (like in figure 2) especially in Fig 3E where it really looks like there might be a reduction in active Ras levels.

We have presented individual data points for Figure 3 (now Figure 5) to illustrate the distribution of the data. It is worth noting that the decrease in active Ras levels observed in cells lacking PTEN or CnrN is not significantly different from that in Ax2 cells.

10. Figure 3: Why are there not error bars on any of the initial conditions (eg Ax2 in 3B). I understand that the other values are normalized to AX2, however if there are 3 independent experiments, each AX2 individual experiment would likely differ from that average.

The values for Ax2 for each independent experiment were set as 100%. Therefore, there are no error bars on any of the controls. We have now explicitly stated this in the figure legends. as: "For each independent experiment, values for basal total or active Ras in Ax2 (B and C), or total or active Ras at time 0 in Ax2, were set as 100%."

11. Supplementary Figure 1: the DNA ladders should be marked properly

Done.

12. Also, for Figures S4, S5, etc., the authors would do well to include some imaging data to supplement these graphs. Additionally, while there is some description of how filopodia, macropinosomes, and pseudopods are quantified, it isn't entirely clear how one distinguishes a macropinosome from a pseudopod. It wasn't clear why there would be such a large change in the size of your pseudopods here compared to what you previously reported (10X larger!, line 233). Without 3D imaging, it is difficult to makes these comparisons. If you are going to include this data, Please elaborate in the methods section how these parameters were scored and measured.

We have representative images of cells displaying pseudopods, filopods, and macropinosomes in Figure S2A (previously S4). In this study, we carefully examined the images over time for projections, as depicted in Figure S2A, to compare their size, lifespan, and frequency. It is worth noting that distinguishing between a macropinosome and a pseudopod can be confusing in some cells, and thus, we refrained from scoring the projections in those instances. While fluorescent markers could potentially aid in visualizing filopods, macropinosomes, and pseudopods using fluorescence microscopy, this would be a focus of our follow-up study based on these initial findings. We have now clarified the method section accordingly as "To avoid discrepancies, we scored cells with projections as shown in Figure S2A. Any cells which had projections that were difficult to distinguish as a macropinosome or a pseudopod, were not scored."

We agree that using 3D imaging would make it easier to compare pseudopods and macropinosomes. However, since we have a lot of comparisons to make among cells, doing a 3D imaging study would take a long time, so we plan to do that as our next project. We have clearly explained in lines 231 – 233 why we might see such a big difference in the size of pseudopods here compared to what we previously reported as "In the previous study, we used cells taken from shaking suspension culture, and in this study, we used cells that had been growing on surfaces in stationary submerged culture. The difference may thus be due to the different culture conditions."

Minor Comments and Typos:

13. There are many run-on sentences in the manuscript, I mentioned several of them below. Line 52: 'phosphatidylinositol3OH -kinases' is not written correctly

The symbols in the manuscript were altered during the PDF conversion process. However, all the letters are correctly written in the original manuscript.

© 2024. Published by The Company of Biologists under the terms of the Creative Commons Attribution License (https://creativecommons.org/licenses/by/4.0/).

14. Line 77: Delete the extra ‘At’
Done (now line 74).

15. Line 146-152 is a run-on sentence. Please break those conclusions up.
We have carefully clarified the text (now 158 – 164).

16. Line 222 cells have “a” slow
Done

17. Line 224 To determine (sentence is a run-on and some of that should be in methods section).
We have now carefully omitted the methods described in the main text. The method section clearly outlines the imaging procedure.

18. Line 306: This increase…(run-on sentence).
We have now split the sentence into two (now line 307).

19. Line 353: In support of….(run-on sentence).
We have now split the sentence into two (now line 355).

20. Fix Veltman 2015 Reference. Biochem Soc Trans (2015) 43 (1): 129–132. PMID: 25619258. It is important to understand the conclusions about macropinocytosis and PIP3 in this paper as well and their primary research papers. They were comparing NC4 cell lines (which grow on bacteria) with the AX2 and 3 lines, which have a mutation that upregulates macropinocytosis and PIP3, thus allowing the cells to grow axenically.
Thank you for catching that mistake. We have now corrected the reference.

Second decision letter

MS ID#: JOCES/2024/262054

MS TITLE: PTEN and the PTEN-like phosphatase CnrN have both distinct and overlapping roles in a Dictyostelium chemorepulsion pathway

AUTHORS: Kristen M. Consalvo, Ramesh Rijal, Steven Beruvides, Ryan Mitchell, Karissa Beauchemin, Danni Collins, Jack Scoggin, Jerome Scott, and Richard Gomer

I am happy to tell you that your manuscript has been accepted for publication in Journal of Cell Science, pending standard publication integrity checks.

Reviewer 1

Advance summary and potential significance to field

Same as in my original review

Comments for the author

The authors have addressed all the critical issues raised by the original manuscript. The revised manuscript is suitable for publication.

Reviewer 2

© 2024. Published by The Company of Biologists under the terms of the Creative Commons Attribution License (https://creativecommons.org/licenses/by/4.0/).

Advance summary and potential significance to field

The authors report observations suggesting that the Dictyostelium PTEN-like phosphatase CnrN has some roles in regulating chemorepulsion in this organism that are distinct from those of PTEN while other roles are redundant. This is interesting from an evolutionary perspective because this CnrN phosphatase is lost in higher eukaryotes, and also because it highlights the usefulness of Dictyostelium to decipher the different roles of PTEN and PIP3 metabolism in biology, roles that have merged in higher eukaryotes and make it more difficult to study process-specific mechanisms.

Comments for the author

The authors appropriately revised their manuscript, this reviewer has no further comments or concerns and recommends publication.

© 2024. Published by The Company of Biologists under the terms of the Creative Commons Attribution License (https://creativecommons.org/licenses/by/4.0/).
